# EF-Feddr: communication-efficient federated learning with Douglas–Rachford splitting and error feedback

**DOI:** 10.3389/frai.2026.1699896

**Published:** 2026-01-28

**Authors:** Jiao Xue, Chundong Wang

**Affiliations:** 1 School of Computer Science and Engineering, Tianjin University of Technology, Tianjin, China; 2 Tianjin Police Institute, Tianjin, China

**Keywords:** communication efficiency, composite optimization, data heterogeneity, error feedback, federated learning, operator splitting

## Abstract

**Introduction:**

Federated learning (FL) is a distributed machine learning paradigm that preserves data privacy and mitigates data silos. Nevertheless, frequent communication between clients and the server often becomes a major bottleneck, restricting training efficiency and scalability.

**Methods:**

To address this challenge, we propose a novel communication-efficient algorithm, **EF-Feddr**, for federated composite optimization, where the objective function includes a potentially non-smooth regularization term and local datasets are non-IID. Our method is built upon the relaxed Douglas–Rachford splitting method and incorporates error feedback (EF)—a widely adopted compression framework—to ensure convergence when biased compression (e.g., top-*k* sparsification) is applied.

**Results:**

Under the partial client participation setting, our theoretical analysis demonstrates that **EF-Feddr** achieves a fast convergence rate of *O*(1/*K*) and a communication complexity of *O*(1/ε^2^). Comprehensive experiments conducted on the FEMNIST and Shakespeare benchmarks, as well as controlled synthetic data, consistently validate the efficacy of **EF-Feddr** across diverse scenarios.

**Discussion:**

The results confirm that the integration of error feedback with the relaxed Douglas–Rachford splitting method in **EF-Feddr** effectively overcomes the convergence degradation typically caused by biased compression, thereby offering a practical and efficient solution for communication-constrained federated learning.

## 1 Introduction

Federated learning (FL) (Konecný et al., 2016; McMahan et al., 2017) is a distributed framework designed to address large-scale learning problems across networks of edge clients. In this paradigm, clients update models locally on their private data, while the server aggregates these updates to refine a shared global model. This collaborative process enables the development of global or personalized models without compromising user privacy (Ezequiel et al., 2022; Saifullah et al., 2024). Despite these advantages, communication between clients and the server remains a critical bottleneck, particularly when the number of participating clients is large, bandwidth is constrained, and the models involve high-dimensional parameters (Bhardwaj et al., 2023; Talwar et al., 2021). Recent efforts to improve the communication efficiency of FL have primarily focused on two directions: (i) reducing the number of communication rounds through partial client participation or increased local computation, and (ii) lowering the number of transmitted bits per round via techniques such as quantization and residual gradient compression. While these strategies effectively cut communication costs, they also introduce additional variance, which may widen the neighborhood around the optimal solution and, in some cases, prevent convergence under biased compression. To mitigate these issues, variance-reduction techniques such as error feedback (EF) are commonly employed. In contrast to traditional distributed training, it is unrealistic to assume that data on each local device are always independent and identically distributed (IID). Prior studies have consistently shown that FL accuracy degrades significantly when faced with non-IID or heterogeneous data (Islam et al., 2024). In this study, we focus on the following federated composite optimization (FCO) problem:


$$\begin{array}{l} \underset{x\in {\mathbb{R}}^{d}}{\min}F\left(x\right)=f\left(x\right)+g\left(x\right)=\frac{1}{n}\sum_{i=1}^{n}f_{i}\left(x\right)+g\left(x\right), \end{array}$$ (1)

where *n* denotes the number of clients, *f*_*i*_ is the local loss function for the *i*-th client, which is *L*-smooth and non-convex, and *g* represents the regularization term, which is proper, closed, convex (possibly non-smooth). As a practical example, consider a collaborative environmental monitoring project in which multiple research institutions aim to analyze sensor data from diverse geographical locations to detect climate change patterns. Due to privacy concerns and proprietary restrictions, however, raw data cannot be shared directly. In this case, enforcing sparse regularization becomes particularly important: although the dataset may contain relatively few observations (e.g., readings from a sparse sensor network Bhardwaj et al., 2022), each observation typically involves a high-dimensional set of features such as temperature, humidity, wind speed, and pollution levels, a combination of factors that further justifies the use of sparse regularization to identify salient features and prevent overfitting.

Operator splitting constitutes a broad class of methods for solving optimization problems of the form (Equation 1). These methods decompose numerically intractable components into simpler subproblems, thereby reducing computational complexity, enhancing efficiency, and enabling modular algorithms that are naturally suited for parallelization. Operator splitting has been successfully applied to a wide range of challenging optimization problems. Among these, the Douglas–Rachford splitting method is particularly well-established due to its enhanced iterative stability and accelerated convergence rate. Furthermore, its update rule decomposes the global composite objective into local proximal steps that can be executed in a fully parallel manner. This structure inherently aligns with the distributed nature of federated learning, facilitating efficient client-side computation while also underpinning the method's enhanced iterative stability. From this perspective, many state-of-the-art FL algorithms can be interpreted within the operator splitting framework (Malekmohammadi et al., 2021). Examples include FedAvg (McMahan et al., 2017), FedProx (Li et al., 2020), FedSplit (Pathak and Wainwright, 2020), and FedDR (Tran-Dinh et al., 2021). However, for the FCO Equation 1, existing FL methods such as FedAvg and its communication-efficient variants are primarily designed for smooth, unconstrained settings $\min_{x\in {\mathbb{R}}^{d}}F\left(x\right)=\frac{1}{n}\sum_{i=1}^{n}f_{i}\left(x\right)$. In non-smooth FL settings, subgradient methods are widely used but suffer from slow convergence (Jhunjhunwala et al., 2022). Although proximal operators offer a more effective alternative with superior convergence properties (Liu et al., 2024), their seamless integration into communication-efficient FL frameworks remains limited. Moreover, while compression techniques effectively reduce communication overhead, they introduce additional variance that can enlarge the solution neighborhood and hinder convergence. Critically, existing communication-efficient methods have predominantly been designed for smooth FL problems, leaving a pronounced combined gap in addressing non-smooth federated composite optimization under compression-induced variance and communication constraints simultaneously. To bridge this multifaceted gap, this study presents EF-Feddr, a communication-efficient FL algorithm that employs the Top-*k* sparsification technique to compress transmitted parameters and reduce communication bits, incorporates an error feedback (Li and Li, 2023) mechanism to mitigate variance introduced by compression, and further integrates the relaxed Douglas–Rachford splitting method (He et al., 2021) along with a proximal operator to accelerate the iterative process while effectively handling the non-smoothness of the global regularization term. This integrated design enables EF-Feddr to be applicable to a wider range of scenarios and constrained settings. Leveraging the Douglas–Rachford envelope, we establish convergence guarantees for EF-Feddr in non-convex FL problems under mild assumptions.

Our contributions are summarized as follows:

- We propose EF-Feddr, an algorithm that combines the relaxed Douglas–Rachford splitting method with error feedback to reduce communication costs between clients and the server without sacrificing accuracy in non-IID settings. In addition, the error feedback mechanism enhances the stability of communication-compressed training in FL.
- We establish theoretical convergence guarantees for EF-Feddr based on the Douglas–Rachford envelope. Specifically, our method achieves a convergence rate of $O\left(\frac{1}{K}\right)$ and a communication complexity of $O\left(\frac{1}{\varepsilon^{2}}\right)$ for non-convex loss functions under partial client participation.
- Through experiments on synthetic datasets, the FEMNIST dataset, and the Shakespeare dataset, we show that EF-Feddr improves accuracy by 3.29%–12.97% over state-of-the-art FL variants, while significantly reducing communication costs compared to uncompressed FedDR.

## 2 Related work

### 2.1 Operator splitting methods

Classical operator splitting methods such as Douglas–Rachford (DR), Forward-Backward (FB), and the Alternating Direction Method of Multipliers (ADMM) have recently been adopted in FL (Godavarthi et al., 2025; Goel et al., 2025). FedAvg (McMahan et al., 2017) can be viewed as an instance of *k*-step FB splitting, while FedProx (Li et al., 2020) extends the backward-backward splitting method. It is another FB variant tailored for regularized FL problems. FedSplit (Pathak and Wainwright, 2020), based on Peaceman-Rachford splitting, aims to identify the correct fixed point for strictly convex FL problems. Its communication-efficient variant, Eco-FedSplit (Khirirat et al., 2022), incorporates error-compensated compression. For the FCO problem, FedDR (Tran-Dinh et al., 2021) integrates a randomized block-coordinate strategy with DR splitting to solve non-convex formulations. FedADMM (Wang et al., 2022) leverages ADMM by applying FedDR to the dual form of the FCO problem, while FedTOP-ADMM (Kant et al., 2022) generalizes FedADMM as the first three-operator method used in FL.

### 2.2 Communication-efficient FL

To address the communication bottleneck in FL (Sun et al., 2024), two categories of compression methods have been widely explored: unbiased compressors (e.g., stochastic quantization Alistarh et al., 2017) and biased compressors (e.g., top-*k* sparsification Khirirat et al., 2018). FedPAQ (Reisizadeh et al., 2020) reduces communication costs through periodic averaging, partial client participation, and quantization. However, this reduction comes at the expense of convergence accuracy, which requires additional training iterations. The authors also analyzed the trade-off between communication overhead and convergence in their experiments. The *z*-SignFedAvg algorithm (Tang et al., 2024), a variant of FedAvg, employs stochastic sign-based compression. It achieves accuracy comparable to uncompressed FedAvg while greatly reducing communication overhead. Building on the lazily aggregated gradient rule and error feedback, (Zhou et al., 2023) proposed two communication-efficient algorithms for non-convex FL: EF-LAG and BiEF-LAG, which adapt both uplink and downlink communications. Similarly, FedSQ (Long et al., 2024) introduces a hybrid approach combining sparsity and quantization to reduce communication costs while enhancing convergence.

### 2.3 Error feedback

In the realm of distributed optimization, it has been noted that employing biased compressors for direct updates may decelerate convergence, deteriorate generalization performance, or even induce divergence (Li and Li, 2023). To counteract these issues, error feedback techniques have been introduced, which can reduce the compression error compared to direct compression. The study (Seide et al., 2014) first proposed this method as a heuristic approach, which is inspired by the idea of Sigma-Delta modulation. EF21 (Richtárik et al., 2021) removes strict assumptions such as bounded gradients and bounded dissimilarity, and can handle arbitrary data heterogeneity among clients, but leads to worse computational complexity. EFSkip (Bao et al., 2025) allows arbitrary data heterogeneity and enjoys linear speedup for significantly improving upon previous results.

## 3 Compressed non-convex FL with error feedback

In this section, we present EF-Feddr, an algorithm that integrates error feedback into the relaxed Douglas–Rachford splitting framework to address the non-convex FCO problem. We begin with a brief introduction to the Douglas–Rachford splitting method, followed by an explanation of how error feedback is incorporated to improve communication efficiency. We then provide the detailed formulation of EF-Feddr and analyze its convergence properties. Main notations are listed in Table 1.

**Table 1:** Summary of main notations.

**Notation**	**Description**
*N*	Number of clients
*n*	Number of sampled clients per round
*d*	Dimension of model parameters
*F*(·)	Global loss function
*f*_*i*_(·)	local loss function of *i*-th client
*g*(·)	Regularizer
*C*(·)	Absolute compressor
*K*	Total number of communication rounds between clients and server
*k*	Index of communication round
*S*_*k*_	Set of sampled clients at *k*-th iteration
λ_*k*_	Relaxation parameter
γ	Step size
$y_{i}^{k}$	Local auxiliary variable at the *i*-th client
$z_{i}^{k}$	Approximate proximal update for optimizing the local loss of *i*-th client
$e_{i}^{k}$	Compression-error accumulator at the *i*-th client
$x_{i}^{k}$	Local model parameters of *i*-th client at *k*-th iteration
*x* ^*k*^	Global model parameters at *k*-th iteration

### 3.1 Problem formulation

The FCO Equation 1 is mathematically equivalent to the consensus optimization problem


$$\begin{array}{l} \begin{array}{l} \underset{x_{1},\ldots ,x_{n}}{\min}F\left(x\right)=f\left(x\right)+g\left(x\right)=\frac{1}{n}\overset{n}{\underset{i=1}{\sum }}f_{i}\left(x_{i}\right)+g\left(x\right)\\ subject tox_{1}=x_{2}=\cdots =x_{n}, \end{array} \end{array}$$ (2)

where the consensus constraint set is *E* = {*x* = (*x*_1_, …, *x*_*n*_)|*x*_1_ = *x*_2_ = ⋯ = *x*_*n*_.}. Let *l*_*E*_ be the indicator function of *E*. With the indicator function, one can treat the constrained problem as unconstrained by moving the constraints into the objective function. Then Equation 1 is obviously equivalent to


$$\begin{array}{l} \min\frac{1}{n}\overset{n}{\underset{i=1}{\sum }}f_{i}\left(x_{i}\right)+g\left(x\right)+l_{𝔼}\left(x\right). \end{array}$$ (3)

The first-order optimality condition is given by 0∈∇*f*(*x*)+∂*g*(*x*)+∂*l*_*E*_(*x*), where ∇*f*(*x*) = [∇*f*_1_(*x*_1_), ..., ∇*f*_*n*_(*x*_*n*_)]. A point x^*^ is a stationary point to Equation 1, if $0\in \nabla f\left(x^{*}\right)+\partial g\left(x^{*}\right)+\partial l_{E}\left(x^{*}\right)$. Additionally, the operator splitting method encompasses a broad range of techniques to effectively address this Equation 3. A key advantage of operator splitting methods is their efficient per-iteration operations, which makes them particularly suitable for large-scale applications due to their lower computational costs (He et al., 2021), among which the DR splitting method is particularly well-known. The iteration equations for the DR splitting method are given by


$$\left\{ \begin{array}{l} y^{k+1}=y^{k}+x^{k}-z^{k+1}\\ z^{k+1}={\mathrm{prox}}_{\gamma f}(y^{k})\\ x^{k+1}={\mathrm{prox}}_{\gamma (g+l_{E})}(2z^{k+1}-y^{k+1}). \end{array}\right.$$ (4)

Given that the DR splitting method often demonstrates favorable and stable convergence behavior in practice, we base our approach on its relaxed variant to solve Equation 1. The detailed application is presented in Section 3.3.

For convenience, we introduce the definitions of the key concepts that will be utilized. For a function *f*, the proximal operator at point *x* with a step size γ>0 is


$$\begin{array}{l} {\mathrm{prox}}_{\gamma f}\left(x\right)=\arg\underset{y}{\min}\left\{f\left(y\right)+\frac{1}{2\gamma }||y-x|{|}^{2}\right\}, \end{array}$$

the Moreau envelope of *f* with a step size γ>0 is


$$\begin{array}{l} M_{\gamma f}\left(x\right)=\underset{y}{\min}\left\{f\left(y\right)+\frac{1}{2\gamma }||y-x|{|}^{2}\right\}, \end{array}$$

the gradient mapping of *f* at point *x* with a step size γ>0 is


$$\begin{array}{l} G_{\gamma f}\left(x\right)=\frac{1}{\gamma }\left(x-{\mathrm{prox}}_{\gamma f}\left(x\right)\right). \end{array}$$

We observe that ∇M_γ*f*_(*x*) = *G*_γ*f*_(*x*) (Liu et al., 2019). Moreover, the proximal operator update $z^{k}={\mathrm{prox}}_{\gamma f}\left(y^{k}\right)$ can be written as


$$\begin{array}{l} z^{k}=y^{k}-\gamma G_{\gamma f}\left(y^{k}\right). \end{array}$$

This representation reveals that the proximal operator update is analogous to taking a gradient step applied to the gradient mapping $G_{\gamma f}\left(y^{k}\right)$ of *f*. For the composite function *F*(*x*) = *f*(*x*)+*g*(*x*), the corresponding gradient mapping is given by


$$\begin{array}{l} G_{\gamma }\left(x\right)=\frac{1}{\gamma }\left(x-{\mathrm{prox}}_{\gamma g}\left(x-\gamma \nabla f\left(x\right)\right)\right). \end{array}$$ (5)

In the context of general non-convex non-smooth problems, the gradient mapping $G_{\gamma }\left(x\right)$ is commonly used to assess convergence (Liu et al., 2024). Specifically, $0\in \nabla f\left(x^{*}\right)+\partial g\left(x^{*}\right)+\partial l_{E}\left(x^{*}\right)$ of Equation 1 is equivalent to $G_{\gamma }\left(x^{*}\right)=0$.

### 3.2 Error feedback

We now define a general class of compressors that will be used throughout this study

Definition 1. (Absolute compressor). A map *C*:ℝ^*d*^ → ℝ^*d*^ is an absolute compressor operator if there exists ν>0 such that, ∀*x*∈ℝ^*d*^, *E*||*x*−*C*(*x*)||^2^ ≤ ν^2^.

Most popular compressors such as the sign compression (Bernstein et al., 2018), the Top-*k* sparsification (Khirirat et al., 2018) and the sparsification together with quantization (Alistarh et al., 2017) are in fact absolute compressors if the full-precision vector has a bounded norm (Khirirat et al., 2022; Sahu et al., 2021).

Error feedback (also known as error compensation) is a popular tool in FL to reduce compression error and improve convergence speed compared to direct compression (Valdeira et al., 2025). Its mechanism shares a fundamental principle with Sigma-Delta modulation in signal processing (Seide et al., 2014). Technically, when transmitting a sequence of vectors, the method incorporates an auxiliary vector that accumulates the compression error at each step. This accumulated error is then added to the current vector before it undergoes compression and transmission (Karimireddy et al., 2019). More specifically, based on the DR splitting method (Equation 4), the update steps of the direct compression scheme are as follows:


$$\begin{array}{l} \begin{array}{l} c^{k+1}=C\left(2z^{k+1}-y^{k+1}\right),\;(direct compression)\\ x^{k+1}={\mathrm{prox}}_{\gamma \left(g+l_{E}\right)}\left(c^{k+1}\right),\;(model update) \end{array} \end{array}$$ (6)

the update steps with error feedback compression are as follows:


$$\begin{array}{l} \begin{array}{l} c^{k+1}=C\left(2z^{k+1}-y^{k+1}+e^{k}\right),\;(error compensation)\\ e^{k+1}=2z^{k+1}-y^{k+1}+e^{k}-c^{k+1},\;(compute the error)\\ x^{k+1}={\mathrm{prox}}_{\gamma \left(g+l_{E}\right)}\left(c^{k+1}\right).\;(model update) \end{array} \end{array}$$ (7)

In direct compression, each vector 2*z*^*k*+1^−*y*^*k*+1^ is individually compressed, and the receiver directly uses its compressed version *C*(2*z*^*k*+1^−*y*^*k*+1^) in place of the original. Conversely, error feedback compression employs a proxy vector *c*^*k*+1^ for 2*z*^*k*+1^−*y*^*k*+1^ that integrates information from prior steps 0, 1, …, *k*. This proxy is refined via an auxiliary vector *e*^*k*+1^, which is iteratively updated and stored to accumulate the compression error at each step.

### 3.3 EF-Feddr algorithm

In this section, we propose the following EF-Feddr algorithm. The details of EF-Feddr are presented in Algorithm 1. Specifically, applying the relaxed DR splitting method (He et al., 2021) to the Equation 3 of Equation 1 in a distributed setting yields the following iterative steps:


$$\{\begin{array}{l} y_{i}^{k+1}=y_{i}^{k}+\lambda \left(x^{k}-z_{i}^{k}\right)\\ z_{i}^{k+1}={\mathrm{prox}}_{\gamma f_{i}}\left(y_{i}^{k+1}\right)\\ x_{i}^{k+1}=2z_{i}^{k+1}-y_{i}^{k+1}\\ x^{k+1}={\mathrm{prox}}_{n\gamma (g+l_{E})}(x_{i}^{k+1}). \end{array}$$

Algorithm 1 EF-Feddr. 
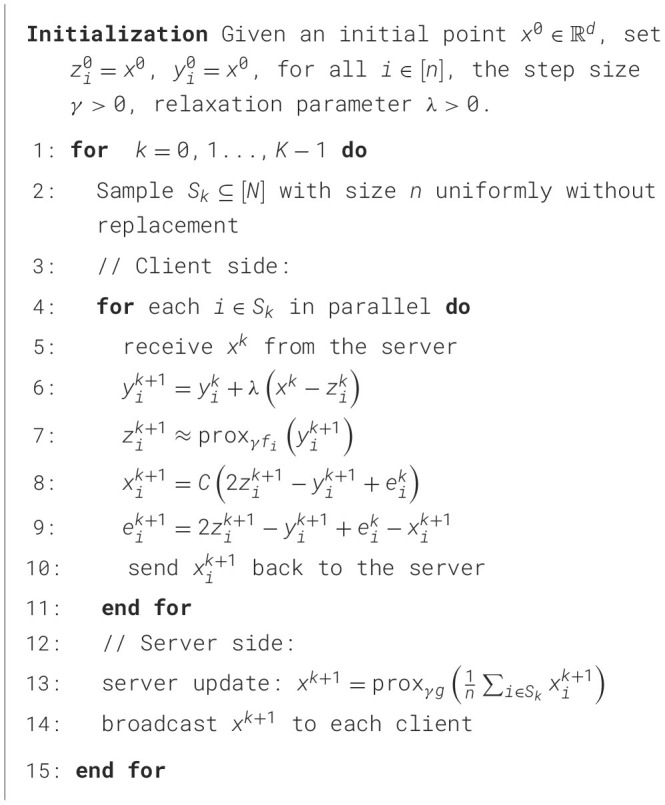

By integrating the error feedback mechanism detailed in Section 3.2, we obtain the EF-Feddr iterative scheme:


$$\left\{ \begin{array}{l} y_{i}^{k+1}=y_{i}^{k}+\lambda \left(x^{k}-z_{i}^{k}\right)\\ z_{i}^{k+1}\approx {\mathrm{prox}}_{\gamma f_{i}}\left(y_{i}^{k+1}\right)\\ x_{i}^{k+1}=C\left(2z_{i}^{k+1}-y_{i}^{k+1}+e_{i}^{k}\right)\\ e_{i}^{k+1}=2z_{i}^{k+1}-y_{i}^{k+1}+e_{i}^{k}-x_{i}^{k+1}\\ x^{k+1}={\mathrm{prox}}_{n\gamma (g+l_{E})}(x_{i}^{k+1}), \end{array}\right.$$ (8)

where λ∈(0, 2) (He et al., 2021) is the relaxation parameter. The variables $y_{i}^{k+1}$, $z_{i}^{k+1}$, $x_{i}^{k+1}$ and $e_{i}^{k+1}$ are updated locally on each client *i*. The key step involves compression and communication: instead of compressing $2z_{i}^{k+1}-y_{i}^{k+1}$ directly, each client compresses the error-compensated vector $2z_{i}^{k+1}-y_{i}^{k+1}+e_{i}^{k}$. The resulting value $x_{i}^{k+1}$ is then sent to the server. Furthermore, to compute the server aggregation *x*^*k*+1^, we have the following conclusion.

Proposition 1. For every *k*≥0, $x^{k+1}={\mathrm{prox}}_{n\gamma \left(g+l_{E}\right)}\left(x_{i}^{k+1}\right)$ in Equation 8 is equal to ${\mathrm{prox}}_{\gamma g}\left(\frac{1}{n}\underset{i\in S_{k}}{\sum }x_{i}^{k+1}\right)$.

Proof. Let $\bar{x}=\frac{1}{n}\underset{i\in S_{k}}{\sum }x_{i}^{k+1}$. Actually, the result of ${\mathrm{prox}}_{n\gamma \left(g+l_{E}\right)}\left(x_{i}^{k+1}\right)$ must have blocks equal to some vector *z* (Mishchenko et al., 2022) such as


z=arg miny{g(y)+12nγ∑i=1n‖y−xik+1‖2}=arg miny{g(y)+12nγ∑i=1n(‖y−x¯‖2+2〈y−x¯,x¯−xik+1〉+‖x¯−xik+1‖2)}=arg miny{g(y)+12nγ[∑i=1n‖y−x¯‖2+2〈y−x¯,nx¯〉−2〈y−x¯,nx¯〉]}=arg miny{g(y)+12γ‖y−x¯‖2}=proxγg(x¯)=proxγg(1n∑i∈Skxik+1).

Thus, we have the server aggregation


$$\begin{array}{l} x^{k+1}={\mathrm{prox}}_{n\gamma \left(g+l_{E}\right)}\left(x_{i}^{k+1}\right)={\mathrm{prox}}_{\gamma g}\left(\frac{1}{n}\sum_{i\in S_{k}}x_{i}^{k+1}\right). \end{array}$$

In Algorithm 1, during round *k*: (1) The clients receive the global model *x*^*k*^ from the server (line 5); (2) A subset of clients *S*_*k*_ is sampled following the sampling scheme described in Section 4. The *i*-th client performs a relaxation step, where λ is the relaxation parameter, computes the proximal local update to obtain the local model $z_{i}^{k+1}$, calculates the compressed local model update $x_{i}^{k+1}$, and updates the local compression error accumulator $e_{i}^{k+1}$ and sends the compressed $x_{i}^{k+1}$ back to the server (line 6–10); (3) The server receives the compressed $x_{i}^{k+1}$ from clients *i*∈*S*_*k*_ and performs a global model update using the averaged compressed local model updates (line 13). Particularly, the relaxation strategy, akin to the inertial extrapolation technique (e.g., the heavy ball method), has broadly accelerated iterative algorithms in convex and non-convex optimization, as the cost per iteration stays basically unchanged (He et al., 2021). For any γ>0, $z_{i}^{k+1}$ serves as an approximation of ${\mathrm{prox}}_{\gamma f_{i}}\left(y_{i}^{k+1}\right)$. The evaluation of prox_γ_*f*__*i*__ can be carried out using several established techniques, such as accelerated GD-type algorithms and local SGD (Parikh et al., 2014; Tran-Dinh et al., 2021). It is worth noting that this algorithm requires *O*(*d*) memory and incurs *O*(*d*) computational overhead per client per round.

## 4 Theoretical results

For analyzing the convergence of Algorithm 1, we consider several basic assumptions and auxiliary results. Our analysis is based on the analytical framework outlined in Tran-Dinh et al. (2021). First, we introduce a proper sampling scheme following Tran-Dinh et al. (2021). Let **p**_1_, …, **p**_*n*_>0 such that for all *i*∈[*N*], $\mathbb{P}\left(i\in \bar{S}\right)={\text{p}}_{i}\leq 1$. Here, $\bar{S}$ is a proper samping scheme of [*N*], and each *S*_*k*_ is an i.i.d. realization of $\bar{S}$. Note that ${\text{p}}_{i}=\underset{S\subseteq \left[N\right],i\in S}{\sum }\mathbb{P}\left(\bar{S}=S\right)$. Define $A_{k}=\sigma \left(S_{0},\ldots ,S_{k}\right)$ as the σ-algebra generated by the sequence *S*_0_, …, *S*_*k*_. This sampling scheme ensures that each client has a significant probability of being updated.

Assumption 1. (*L*-Smoothness). All local functions *f*_*i*_(·) are *L*-smooth, if


$$\begin{array}{l} \forall x,y,||\nabla f_{i}\left(x\right)-\nabla f_{i}\left(y\right)||\leq L||x-y||. \end{array}$$

Assumption 2. (Boundedness from below). *F*(·) given in (1) is bounded below, that is, $F^{*}=\underset{x\in {\mathbb{R}}^{d}}{\inf}F\left(x\right)>-\infty .$

In non-convex FL optimization, Assumptions 1 and 2 are standard. Assumption 2 guarantees that Equation 1 is well-defined and is independent of the choice of algorithms. We first present three useful lemmas that will be instrumental in proving our main theorem.

Lemma 1. Let $\left\{\left(y_{i}^{k},z_{i}^{k},x_{i}^{k},e_{i}^{k},x^{k}\right)\right\}$ be generated by Algorithm 1, for all *i*∈*S*_*k*_, λ>0, β_1_>0 and γ>0, we have


$$\begin{array}{l} ||x^{k}-z_{i}^{k}|{|}^{2}\leq \frac{2\left(\gamma^{2}L^{2}+1\right)}{\lambda^{2}}[\left(1+\beta_{1}\right)||z_{i}^{k+1}-z_{i}^{k}|{|}^{2}\\ \;+2\left(1+\frac{1}{\beta_{1}}\right)\left(||m_{i}^{k+1}|{|}^{2}+||m_{i}^{k}|{|}^{2}\right)]. \end{array}$$ (9)

Proof.

For the relation $z_{i}^{k+1}\approx {\mathrm{prox}}_{\gamma f_{i}}\left(y_{i}^{k+1}\right)$, where the approximation error satisfies $||z_{i}^{k+1}-{\mathrm{prox}}_{\gamma f_{i}}\left(y_{i}^{k+1}\right)||\leq \varepsilon_{i}^{k}$ with a given accuracy $\varepsilon_{i}^{k}\geq 0$, we introduce auxiliary variables $w_{i}^{0}$ and $w_{i}^{k+1}$ for *i*∈[*n*] to analyze the convergence of Algorithm 1,


$$\begin{array}{l} w_{i}^{0}={\mathrm{prox}}_{\gamma f_{i}}(y_{i}^{0}),\\ w_{i}^{k+1}=\{\begin{array}{ll} {\mathrm{prox}}_{\gamma f_{i}}(y_{i}^{k+1}) & \text{if }i\in S_{k}\\ w_{i}^{k} & \text{if }i\notin S_{k} \end{array},\\ z_{i}^{k}=w_{i}^{k}+m_{i}^{k},\text{ where }\|m_{i}^{k}\|\leq \varepsilon_{i}^{k}. \end{array}$$ (10)

Here, $m_{i}^{k}$ denotes the vector of errors associated with the approximations of the proximal operator, and $w_{i}^{k+1}$ serves as an accurate computation to ${\mathrm{prox}}_{\gamma f_{i}}\left(y_{i}^{k+1}\right)$. Note that when $i\notin S_{k}$, we have $z_{i}^{k+1}=z_{i}^{k}$ and $w_{i}^{k+1}=w_{i}^{k}$, which implies $\left||m_{i}^{k+1}||=||z_{i}^{k+1}-w_{i}^{k+1}||=||m_{i}^{k}||=||z_{i}^{k}-w_{i}^{k}|\right|$. From Equation 10 (Atenas, 2025), we have


$$\begin{array}{l} y_{i}^{k}=w_{i}^{k}+\gamma \nabla f_{i}\left(w_{i}^{k}\right). \end{array}$$ (11)

Then, using the update rule for $y_{i}^{k+1}$ in Algorithm 1, we get $x^{k}-z_{i}^{k}=\frac{1}{\lambda }\left(y_{i}^{k+1}-y_{i}^{k}\right)=\frac{1}{\lambda }\left(w_{i}^{k+1}-w_{i}^{k}\right)+\frac{\gamma }{\lambda }\left(\nabla f_{i}\left(w_{i}^{k+1}\right)-\nabla f_{i}\left(w_{i}^{k}\right)\right).$ Using Young's inequality $||a_{1}+a_{2}|{|}^{2}\leq \left(1+\beta \right)||a_{1}|{|}^{2}+\left(1+\frac{1}{\beta }\right)||a_{2}|{|}^{2}$, and the *L*-smoothness of *f*_*i*_, we bound $||x^{k}-z_{i}^{k}|{|}^{2}$ for any β_1_>0 and $i\in S_{k}$ as follows


$$\begin{array}{l} {\left\|x^{k}-z_{i}^{k}\right\|}^{2}={\left\|\frac{1}{\lambda }(w_{i}^{k+1}-w_{i}^{k})+\frac{\gamma }{\lambda }(\nabla f_{i}(w_{i}^{k+1})-\nabla f_{i}(w_{i}^{k}))\right\|}^{2}\\ \;\leq \frac{2}{\lambda^{2}}{\left\|w_{i}^{k+1}-w_{i}^{k}\right\|}^{2}+\frac{2\gamma^{2}}{\lambda^{2}}{\left\|\left(\nabla f_{i}(w_{i}^{k+1})-\nabla f_{i}(w_{i}^{k})\right)\right\|}^{2}\\ \;\leq \frac{2}{\lambda^{2}}{\left\|w_{i}^{k+1}-w_{i}^{k}\right\|}^{2}+\frac{2\gamma^{2}L^{2}}{\lambda^{2}}{\left\|\left(w_{i}^{k+1}-w_{i}^{k}\right)\right\|}^{2}\\ \;=\frac{2(\gamma^{2}L^{2}+1)}{\lambda^{2}}{\left\|z_{i}^{k+1}-m_{i}^{k+1}-z_{i}^{k}+m_{i}^{k}\right\|}^{2}\\ \;\leq \frac{2(\gamma^{2}L^{2}+1)}{\lambda^{2}}[(1+\beta_{1})\|z_{i}^{k+1}-z_{i}^{k}{\|}^{2}+2(1\\ \;+\frac{1}{\beta_{1}})(\|m_{i}^{k+1}{\|}^{2}+\|m_{i}^{k}{\|}^{2})], \end{array}$$

which proves (9).

We then establish the relationship between $\overset{n}{\underset{i=1}{\sum }}||x^{k}-z_{i}^{k}|{|}^{2}$ and the squared norm of the gradient mapping $||G_{\gamma }\left(x^{k}\right)|{|}^{2}$.

Lemma 2. Let $\left\{\left(y_{i}^{k},z_{i}^{k},x_{i}^{k},e_{i}^{k},x^{k},w_{i}^{k}\right)\right\}$ be generated by Algorithm 1 and Equation 10, and the gradient mapping $G_{\gamma }$ be defined by (5). Then, for any λ>0, β_2_>0, and γ>0, we have


$$\begin{array}{l} \begin{array}{l} ||G_{\gamma }\left(x^{k}\right)|{|}^{2}\leq \frac{2{\left(1+\gamma L\right)}^{2}}{n\gamma^{2}}\overset{n}{\underset{i=1}{\sum }}[\left(1+\beta_{2}\right)||z_{i}^{k}-x^{k}|{|}^{2}\\ \;+\left(1+\frac{1}{\beta_{2}}\right)||m_{i}^{k}|{|}^{2}]+\frac{2}{n\gamma^{2}}\overset{n}{\underset{i=1}{\sum }}||e_{i}^{k-1}-e_{i}^{k}|{|}^{2}. \end{array} \end{array}$$ (12)

Proof. From the update of $x_{i}^{k+1}$, $e_{i}^{k+1}$ in Algorithm 1 and (11), we have


$$\begin{array}{l} \begin{array}{l} \frac{1}{n}\overset{n}{\underset{i=1}{\sum }}x_{i}^{k}=\frac{1}{n}\overset{n}{\underset{i=1}{\sum }}\left(2z_{i}^{k}-y_{i}^{k}+e_{i}^{k-1}-e_{i}^{k}\right)\\ \;=\frac{1}{n}\overset{n}{\underset{i=1}{\sum }}\left(2z_{i}^{k}-w_{i}^{k}-\gamma \nabla f_{i}\left(w_{i}^{k}\right)+e_{i}^{k-1}-e_{i}^{k}\right). \end{array} \end{array}$$ (13)

From the update rule of *x*^*k*^ in Algorithm 1, the definition of $G_{\gamma }\left(x\right)$, the non-expansive property of prox_γ*g*_, and the fact $\nabla f\left(x^{k}\right)=\frac{1}{n}\overset{n}{\underset{i=1}{\sum }}\nabla f_{i}\left(x^{k}\right)$, we obtain that


$$\begin{array}{l} \left\|G_{\gamma }(x^{k})\|=\frac{1}{\gamma }\|x^{k}-{\mathrm{prox}}_{\gamma g}(x^{k}-\gamma \nabla f(x^{k}))\right\|\\ \;=\frac{1}{\gamma }\|{\mathrm{prox}}_{\gamma g}(\frac{1}{n}\sum_{i=1}^{n}x_{i}^{k})-{\mathrm{prox}}_{\gamma g}(x^{k}-\gamma \nabla f(x^{k}))\|\\ \;\leq \frac{1}{\gamma }\|\frac{1}{n}\sum_{i=1}^{n}x_{i}^{k}-x^{k}+\gamma \nabla f(x^{k})\|\;\\ \;=\frac{1}{n\lambda }||\sum_{i=1}^{n}\left[(2z_{i}^{k}-w_{i}^{k}-x^{k})+\gamma (\nabla f_{i}(x^{k})-\nabla f_{i}(w_{i}^{k})\right)\\ \;+e_{i}^{k-1}-e_{i}^{k}]\|. \end{array}$$

By applying the *L*-smoothness of *f*_*i*_ and the Young's inequality stated in Lemma 1, for any β_2_>0 we deduce that


$$\begin{array}{l} ||G_{\gamma }\left(x^{k}\right)|{|}^{2}\\ \leq \frac{1}{n^{2}\gamma^{2}}{\left[\overset{n}{\underset{i=1}{\sum }}\left(||2z_{i}^{k}-w_{i}^{k}-x^{k}||+\gamma L||z_{i}^{k}-{\bar{x}}^{k}||+||e_{i}^{k-1}-e_{i}^{k}||\right)\right]}^{2}\\ \leq \frac{1}{n\gamma^{2}}\overset{n}{\underset{i=1}{\sum }}{\left(||2z_{i}^{k}-w_{i}^{k}-x^{k}||+\gamma L||x^{k}-w_{i}^{k}||+||e_{i}^{k-1}-e_{i}^{k}||\right)}^{2}\\ \leq \frac{1}{n\gamma^{2}}\overset{n}{\underset{i=1}{\sum }}{\left[\left(1+\gamma L\right)||z_{i}^{k}-x^{k}||+\left(1+\gamma L\right)||m_{i}^{k}||+||e_{i}^{k-1}-e_{i}^{k}||\right]}^{2}\\ \leq \frac{1}{n\gamma^{2}}{\left(1+\gamma L\right)}^{2}\overset{n}{\underset{i=1}{\sum }}[2\left(1+\beta_{2}\right)||z_{i}^{k}-x^{k}|{|}^{2}+2\left(1+\frac{1}{\beta_{2}}\right)||m_{i}^{k}|{|}^{2}\\ +\frac{2}{{\left(1+\gamma L\right)}^{2}}||e_{i}^{k-1}-e_{i}^{k}|{|}^{2}]\\ \leq \frac{2{\left(1+\gamma L\right)}^{2}}{n\gamma^{2}}\overset{n}{\underset{i=1}{\sum }}[\left(1+\beta_{2}\right)||z_{i}^{k}-x^{k}|{|}^{2}+\left(1+\frac{1}{\beta_{2}}\right)||m_{i}^{k}|{|}^{2}\\ +\frac{1}{{\left(1+\gamma L\right)}^{2}}||e_{i}^{k-1}-e_{i}^{k}|{|}^{2}], \end{array}$$

which proves (12).

Lemma 3. Let $\left\{\left(y_{i}^{k},z_{i}^{k},x_{i}^{k},e_{i}^{k},x^{k}\right)\right\}$ be generated by Algorithm 1. Suppose that Assumptions 1 and 2 hold, and we define the Lyapunov function


$$\begin{array}{l} V^{k}\left(x^{k}\right)=g\left(x^{k}\right)+\frac{1}{n}\overset{n}{\underset{i=1}{\sum }}[f_{i}\left(z_{i}^{k}\right)+\langle \nabla f_{i}\left(z_{i}^{k}\right),x^{k}-z_{i}^{k}\rangle \\ \;+\frac{1}{2\gamma }||x^{k}-z_{i}^{k}|{|}^{2}], \end{array}$$

then by choosing


$$\begin{array}{l} 0<\gamma <\frac{\sqrt{{\left(1-\frac{\lambda }{4}\right)}^{2}-\lambda^{2}\beta_{4}\left(4\beta_{4}+1\right)}-\frac{\lambda }{4}}{L\left(2\lambda \beta_{4}+1\right)}\;\mathrm{and}\\ 0<\lambda <\frac{\min\left\{\sqrt{4\beta_{4}+\frac{17}{16}}-\frac{1}{4},2\right\}}{4\beta_{4}+1}, \end{array}$$

and for any ε_1_, β_1_, β_4_>0, we have


$$\begin{array}{l} 𝔼\left[V^{k+1}\left(x^{k+1}\right)|A_{k-1}\right]\leq V^{k}\left(x^{k}\right)-\frac{\pi }{2n}\overset{n}{\underset{i=1}{\sum }}||x^{k}-z_{i}^{k}|{|}^{2}+\frac{4\varepsilon_{1}}{\gamma }\nu^{2}\\ \;+\frac{1}{n}\overset{n}{\underset{i=1}{\sum }}\left(\delta_{1}{\left(\varepsilon_{i}^{k}\right)}^{2}+\delta_{2}{\left(\varepsilon_{i}^{k+1}\right)}^{2}\right), \end{array}$$

where


$$\begin{array}{l} \pi =\frac{\text{p}\lambda \left[2-\lambda \left(1+L\gamma \right)-2L^{2}\gamma^{2}-4\lambda \beta_{4}\left(1+L^{2}\gamma^{2}\right)\right]}{2\gamma \left(1+\beta_{1}\right)\left(\gamma^{2}L^{2}+1\right)},\\ \delta_{1}=\frac{2{\left(1+\gamma L\right)}^{2}}{\gamma \beta_{4}\lambda^{2}}+\frac{\left[2-\lambda \left(1+L\gamma \right)-2L^{2}\gamma^{2}-4\lambda \beta_{4}\left(1+L^{2}\gamma^{2}\right)\right]}{\lambda \gamma \beta_{1}},\\ \delta_{2}=\delta_{1}+\frac{\left(1+\gamma^{2}L^{2}\right)}{\gamma }. \end{array}$$

Proof. Given the definition ${\bar{x}}^{k}=\frac{1}{n}\overset{n}{\underset{i=1}{\sum }}x_{i}^{k}$, the update rule $x^{k+1}={\mathrm{prox}}_{\gamma g}\left({\bar{x}}^{k+1}\right)$ in Algorithm 1 (hence $\frac{{\bar{x}}^{k+1}-x^{k+1}}{\gamma }\in \partial g\left(x^{k+1}\right)$), and the convexity of *g*, we obtain the following inequality


$$\begin{array}{l} g\left(x^{k+1}\right)\leq g\left(x^{k}\right)-\frac{1}{\gamma }||x^{k+1}-x^{k}|{|}^{2}+\frac{1}{\gamma }\langle {\bar{x}}^{k+1}-x^{k},x^{k+1}-x^{k}\rangle . \end{array}$$ (14)

Combining Equations 10 and 11, we obtain


$$\begin{array}{l} z_{i}^{k+1}+\gamma \nabla f_{i}(z_{i}^{k+1})\;=w_{i}^{k+1}+\gamma \nabla f_{i}(w_{i}^{k+1})+m_{i}^{k+1}+\gamma (\nabla f_{i}(z_{i}^{k+1})\\ \;-\nabla f_{i}(w_{i}^{k+1}))\\ \;=y_{i}^{k+1}+m_{i}^{k+1}+\gamma (\nabla f_{i}(z_{i}^{k+1})-\nabla f_{i}(w_{i}^{k+1})). \end{array}$$ (15)

Next, using the update rules for $x_{i}^{k+1}$ and $e_{i}^{k+1}$ in Algorithm 1, we have


$$\begin{array}{l} {\bar{x}}^{k+1}=\frac{1}{n}\overset{n}{\underset{i=1}{\sum }}x_{i}^{k+1}=\frac{1}{n}\overset{n}{\underset{i=1}{\sum }}\left(C\left(2z_{i}^{k+1}-y_{i}^{k+1}+e_{i}^{k}\right)\right)\\ \;=\frac{1}{n}\overset{n}{\underset{i=1}{\sum }}\left(2z_{i}^{k+1}-y_{i}^{k+1}+e_{i}^{k}-e_{i}^{k+1}\right). \end{array}$$ (16)

In order to establish the descent property of the Lyapunov function *V*^*k*+1^(*x*^*k*+1^), its second term is expanded and rearranged as follows


$$\begin{array}{l} \frac{1}{n}\overset{n}{\underset{i=1}{\sum }}\left[f_{i}\left(z_{i}^{k+1}\right)+\langle \nabla f_{i}\left(z_{i}^{k+1}\right),x^{k+1}-z_{i}^{k+1}\rangle +\frac{1}{2\gamma }||x^{k+1}-z_{i}^{k+1}|{|}^{2}\right]\\ =\frac{1}{n}\overset{n}{\underset{i=1}{\sum }}\left[f_{i}\left(z_{i}^{k+1}\right)+\langle \nabla f_{i}\left(z_{i}^{k+1}\right),x^{k}-z_{i}^{k+1}+x^{k+1}-x^{k}\rangle \right]\\ \;+\frac{1}{2\gamma n}\overset{n}{\underset{i=1}{\sum }}||x^{k}-z_{i}^{k+1}+x^{k+1}-x^{k}|{|}^{2}\\ =\frac{1}{n}\overset{n}{\underset{i=1}{\sum }}\left[f_{i}\left(z_{i}^{k+1}\right)+\langle \nabla f_{i}\left(z_{i}^{k+1}\right),x^{k}-z_{i}^{k+1}\rangle +\frac{1}{2\gamma }||x^{k}-z_{i}^{k+1}|{|}^{2}\right]\\ \;+\frac{1}{n\gamma }\overset{n}{\underset{i=1}{\sum }}\langle x^{k}-2z_{i}^{k+1}+\left(z_{i}^{k+1}+\gamma \nabla f_{i}\left(z_{i}^{k+1}\right)\right),x^{k+1}-x^{k}\rangle \\ +\frac{1}{2\gamma }||x^{k+1}-x^{k}|{|}^{2}\\ \overset{(15)}{=}\frac{1}{n}\overset{n}{\underset{i=1}{\sum }}\left[f_{i}\left(z_{i}^{k+1}\right)+\langle \nabla f_{i}\left(z_{i}^{k+1}\right),x^{k}-z_{i}^{k+1}\rangle +\frac{1}{2\gamma }||x^{k}-z_{i}^{k+1}|{|}^{2}\right]\\ \;+\frac{1}{n\gamma }\overset{n}{\underset{i=1}{\sum }}\langle x^{k}-2z_{i}^{k+1}+y_{i}^{k+1},x^{k+1}-x^{k}\rangle +\frac{1}{2\gamma }||x^{k+1}-x^{k}|{|}^{2}\\ \;+\frac{1}{n\gamma }\overset{n}{\underset{i=1}{\sum }}\langle m_{i}^{k+1}+\gamma \left(\nabla f_{i}\left(z_{i}^{k+1}\right)-\nabla f_{i}\left(w_{i}^{k+1}\right)\right),x^{k+1}-x^{k}\rangle \\ \overset{\left(16\right)}{=}\frac{1}{n}\overset{n}{\underset{i=1}{\sum }}\left[f_{i}\left(z_{i}^{k+1}\right)+\langle \nabla f_{i}\left(z_{i}^{k+1}\right),x^{k}-z_{i}^{k+1}\rangle +\frac{1}{2\gamma }||x^{k}-z_{i}^{k+1}|{|}^{2}\right]\\ \;+\frac{1}{\gamma }\langle x^{k}-{\bar{x}}^{k+1}+\frac{1}{n}\overset{n}{\underset{i=1}{\sum }}\left(e_{i}^{k}-e_{i}^{k+1}\right),x^{k+1}-x^{k}\rangle +\frac{1}{2\gamma }||x^{k+1}\\ -x^{k}|{|}^{2}+\frac{1}{n\gamma }\overset{n}{\underset{i=1}{\sum }}\langle m_{i}^{k+1}+\gamma \left(\nabla f_{i}\left(z_{i}^{k+1}\right)-\nabla f_{i}\left(w_{i}^{k+1}\right)\right),x^{k+1}-x^{k}\rangle . \end{array}$$ (17)

Here, Equation 15 is used to separate the term $y_{i}^{k+1}$ from the approximation error $m_{i}^{k+1}$, while Equation 16 expresses $2z_{i}^{k+1}-y_{i}^{k+1}$ in terms of the average vector ${\bar{x}}^{k+1}$ and the accumulated compression errors $e_{i}^{k+1}$ and $e_{i}^{k}$. Then, by combining Equations 14, 17 and using the definition of *V*^*k*+1^(*x*^*k*+1^), we obtain that


$$\begin{array}{l} V^{k+1}(x^{k+1})\;\leq g(x^{k})+\frac{1}{n}\sum_{i=1}^{n}[f_{i}(z_{i}^{k+1})+\langle \nabla f_{i}(z_{i}^{k+1}),x^{k}-z_{i}^{k+1}\rangle \\ \;+\frac{1}{2\gamma }\|x^{k}-z_{i}^{k+1}{\|}^{2}]\\ \;+\frac{1}{n\gamma }\sum_{i=1}^{n}\langle e_{i}^{k}-e_{i}^{k+1},x^{k+1}-x^{k}\rangle -\frac{1}{2\gamma }\|x^{k+1}-x^{k}{\|}^{2}\\ \;+\frac{1}{n\gamma }\sum_{i=1}^{n}\langle m_{i}^{k+1}+\gamma (\nabla f_{i}(z_{i}^{k+1})-\nabla f_{i}(w_{i}^{k+1})),x^{k+1}\\ \;-x^{k}\rangle \;. \end{array}$$ (18)

To bound the third term on the right-hand side of Equation 18, we employ the inequality $2\langle a_{1},a_{2}\rangle \leq \varepsilon_{1}||a_{1}|{|}^{2}+\frac{1}{\varepsilon_{1}}||a_{2}|{|}^{2}$ (for any ε_1_>0) as follows


$$\begin{array}{l} \frac{1}{n\gamma }\overset{n}{\underset{i=1}{\sum }}\langle e_{i}^{k}-e_{i}^{k+1},x^{k+1}-x^{k}\rangle \\ \leq \frac{1}{n\gamma }\overset{n}{\underset{i=1}{\sum }}\left[\varepsilon_{1}||e_{i}^{k}-e_{i}^{k+1}|{|}^{2}+\frac{1}{\varepsilon_{1}}||x^{k+1}-x^{k}|{|}^{2}\right]\\ \leq \frac{1}{n\gamma }\overset{n}{\underset{i=1}{\sum }}\left[2\varepsilon_{1}||e_{i}^{k}|{|}^{2}+2\varepsilon_{1}||e_{i}^{k+1}|{|}^{2}\right]\\ \;+\frac{1}{\gamma \varepsilon_{1}}||x^{k+1}-x^{k}|{|}^{2}\\ \leq \frac{2\varepsilon_{1}}{n\gamma }\overset{n}{\underset{i=1}{\sum }}\left[||e_{i}^{k}|{|}^{2}+||e_{i}^{k+1}|{|}^{2}\right]\\ \;+\frac{1}{\gamma \varepsilon_{1}}||x^{k+1}-x^{k}|{|}^{2}. \end{array}$$ (19)

For $i\notin S_{k}$, we have $w_{i}^{k+1}=w_{i}^{k}$. Applying Young's inequality stated in Lemma 1 with any β_3_>0, we can evaluate the five term on the right-hand side of Equation 18 as follows


$$\begin{array}{l} \frac{1}{n\gamma }\overset{n}{\underset{i=1}{\sum }}\langle m_{i}^{k+1}+\gamma \left(\nabla f_{i}\left(z_{i}^{k+1}\right)-\nabla f_{i}\left(w_{i}^{k+1}\right)\right),x^{k+1}-x^{k}\rangle \\ \leq \frac{1}{2n\gamma }\overset{n}{\underset{i=1}{\sum }}[\frac{1}{\beta_{3}}||m_{i}^{k+1}+\gamma \left(\nabla f_{i}\left(z_{i}^{k+1}\right)-\nabla f_{i}\left(w_{i}^{k+1}\right)\right)|{|}^{2}+\beta_{3}||x^{k+1}\\ -x^{k}|{|}^{2}]\\ \leq \frac{1}{n\gamma \beta_{3}}\overset{n}{\underset{i=1}{\sum }}\left[||m_{i}^{k+1}|{|}^{2}+\gamma^{2}\overset{n}{\underset{i=1}{\sum }}||\nabla f_{i}\left(x_{i}^{k+1}\right)-\nabla f_{i}\left(z_{i}^{k+1}\right)|{|}^{2}\right]\\ +\frac{\beta_{3}}{2\gamma }||x^{k+1}-x^{k}|{|}^{2}\\ \leq \frac{\left(1+\gamma^{2}L^{2}\right)}{n\gamma \beta_{3}}\left[\underset{i\notin S_{k}}{\sum }||m_{i}^{k}|{|}^{2}+\underset{i\in S_{k}}{\sum }||m_{i}^{k+1}|{|}^{2}\right]+\frac{\beta_{3}}{2\gamma }||x^{k+1}-x^{k}|{|}^{2}. \end{array}$$ (20)

To streamline the notation, denote


$$\begin{array}{l} {\text{Ψ}}_{k+1}=-\frac{1}{\gamma }\left(\frac{1}{2}-\frac{1}{\varepsilon_{1}}-\frac{\beta_{3}}{2}\right)||x^{k+1}-x^{k}|{|}^{2}\\ \;+\frac{2\varepsilon_{1}}{n\gamma }\overset{n}{\underset{i=1}{\sum }}\left[||e_{i}^{k}|{|}^{2}+||e_{i}^{k+1}|{|}^{2}\right]\\ \;+\frac{\left(1+\gamma^{2}L^{2}\right)}{n\gamma \beta_{3}}\left[\underset{i\notin S_{k}}{\sum }||m_{i}^{k}|{|}^{2}+\underset{i\in S_{k}}{\sum }||m_{i}^{k+1}|{|}^{2}\right], \end{array}$$ (21)

and substituting Equations 19 and 20 into Equations 18, we obtain an expanded expression for *V*^*k*+1^. Differentiating between the active client set $S_{k}$ and the inactive set, and employing the *L*- smoothness of *f*_*i*_ (i.e., $f_{i}\left(z_{i}^{k+1}\right)\leq f_{i}\left(z_{i}^{k}\right)+\langle \nabla f_{i}\left(z_{i}^{k}\right),z_{i}^{k+1}-z_{i}^{k}\rangle +\frac{L}{2}||z_{i}^{k+1}-z_{i}^{k}|{|}^{2}$), we have


$$\begin{array}{l} V^{k+1}\left(x^{k+1}\right)\leq g\left(x^{k}\right)+\frac{1}{n}\overset{n}{\underset{i=1}{\sum }}[f_{i}\left(z_{i}^{k+1}\right)+\langle \nabla f_{i}\left(z_{i}^{k+1}\right),x^{k}-z_{i}^{k+1}\rangle \\ \;+\frac{1}{2\gamma }||x^{k}-z_{i}^{k+1}|{|}^{2}]+{\text{Ψ}}_{k+1}\\ \;\left(\text{by the fact that only}i\in S_{k}\text{ perform update}\right)\\ \;=g\left(x^{k}\right)+\frac{1}{n}\underset{i\in S_{k}}{\sum }f_{i}\left(z_{i}^{k+1}\right)+\frac{1}{n}\underset{i\in S_{k}}{\sum }\langle \nabla f_{i}\left(z_{i}^{k+1}\right),z_{i}^{k}-z_{i}^{k+1}\rangle \\ \;+\frac{1}{n}\underset{i\in S_{k}}{\sum }\langle \nabla f_{i}\left(z_{i}^{k+1}\right),x^{k}-z_{i}^{k}\rangle +\frac{1}{2n\gamma }\underset{i\in S_{k}}{\sum }||x^{k}-z_{i}^{k+1}|{|}^{2}\\ \;+\frac{1}{n}\underset{i\notin S_{k}}{\sum }f_{i}\left(z_{i}^{k}\right)+\frac{1}{n}\underset{i\notin S_{k}}{\sum }\langle \nabla f_{i}\left(z_{i}^{k}\right),x^{k}-z_{i}^{k}\rangle \\ \;+\frac{1}{2n\gamma }\underset{i\notin S_{k}}{\sum }||x^{k}-z_{i}^{k}|{|}^{2}+{\text{Ψ}}_{k+1}\\ \;\left(\text{by the}L-\text{smoothness of }f_{i}\right)\\ \leq g\left(x^{k}\right)+\frac{1}{n}\underset{i\in S_{k}}{\sum }f_{i}\left(z_{i}^{k}\right)+\frac{L}{2n}\underset{i\in S_{k}}{\sum }||z_{i}^{k+1}-z_{i}^{k}|{|}^{2}\\ \;+\frac{1}{n}\underset{i\in S_{k}}{\sum }\langle \nabla f_{i}\left(z_{i}^{k+1}\right),x^{k}-z_{i}^{k}\rangle +\frac{1}{2n\gamma }\underset{i\in S_{k}}{\sum }||x^{k}-z_{i}^{k+1}|{|}^{2}\\ \;+\frac{1}{n}\underset{i\notin S_{k}}{\sum }f_{i}\left(z_{i}^{k}\right)+\frac{1}{n}\underset{i\notin S_{k}}{\sum }\langle \nabla f_{i}\left(z_{i}^{k}\right),x^{k}-z_{i}^{k}\rangle \\ +\frac{1}{2n\gamma }\underset{i\notin S_{k}}{\sum }||x^{k}-z_{i}^{k}|{|}^{2}+{\text{Ψ}}_{k+1}\\ =g\left(x^{k}\right)+\frac{1}{n}\overset{n}{\underset{i=1}{\sum }}f_{i}\left(z_{i}^{k}\right)+\frac{1}{n}\overset{n}{\underset{i=1}{\sum }}\langle \nabla f_{i}\left(z_{i}^{k}\right),x^{k}-z_{i}^{k}\rangle \\ +\frac{L}{2n}\underset{i\in S_{k}}{\sum }||z_{i}^{k+1}-z_{i}^{k}|{|}^{2}\\ +\frac{1}{2n\gamma }\underset{i\in S_{k}}{\sum }||x^{k}-z_{i}^{k+1}|{|}^{2}+\frac{1}{n}\underset{i\in S_{k}}{\sum }\langle \nabla f_{i}\left(z_{i}^{k+1}\right)-\nabla f_{i}\left(z_{i}^{k}\right),x^{k}-z_{i}^{k}\rangle \\ +\frac{1}{2n\gamma }\underset{i\notin S_{k}}{\sum }||x^{k}-z_{i}^{k}|{|}^{2}+{\text{Ψ}}_{k+1}. \end{array}$$ (22)

Next, applying the square-norm expansion


$$\begin{array}{l} ||x^{k}-z_{i}^{k+1}|{|}^{2}=||x^{k}-z_{i}^{k}|{|}^{2}+2\langle x^{k}-z_{i}^{k},z_{i}^{k}-z_{i}^{k+1}\rangle +||z_{i}^{k}\\ \;-z_{i}^{k+1}|{|}^{2}. \end{array}$$

For non-updated clients $i\notin S_{k}$, the local variable remains unchanged, i.e., $z_{i}^{k+1}=z_{i}^{k}$. Substituting these relations into the original expression gives


$$\begin{array}{l} \frac{1}{2n\gamma }\underset{i\in S_{k}}{\sum }||x^{k}-z_{i}^{k+1}|{|}^{2}+\frac{1}{2n\gamma }\underset{i\notin S_{k}}{\sum }||x^{k}-z_{i}^{k}|{|}^{2}\\ =\frac{1}{2n\gamma }\overset{n}{\underset{i=1}{\sum }}||x^{k}-z_{i}^{k}|{|}^{2}+\frac{1}{2n\gamma }\underset{i\in S_{k}}{\sum }[2\langle x^{k}-z_{i}^{k},z_{i}^{k}-z_{i}^{k+1}\rangle \\ +||z_{i}^{k}-z_{i}^{k+1}|{|}^{2}], \end{array}$$

Inserting the reorganized expression into the expansion of *V*^*k*+1^(*x*^*k*+1^) and collecting common terms gives


$$\begin{array}{l} \begin{array}{l} V^{k+1}\left(x^{k+1}\right)=V^{k}\left(x^{k}\right)+\frac{1}{n}\underset{i\in S_{k}}{\sum }\langle \nabla f_{i}\left(z_{i}^{k+1}\right)-\nabla f_{i}\left(z_{i}^{k}\right),x^{k}-z_{i}^{k}\rangle \\ \;+\frac{1}{n\gamma }\underset{i\in S_{k}}{\sum }\langle z_{i}^{k+1}-z_{i}^{k},z_{i}^{k}-x^{k}\rangle \\ \;+\frac{1+L\gamma }{2n\gamma }\underset{i\in S_{k}}{\sum }||z_{i}^{k+1}-z_{i}^{k}|{|}^{2}+{\text{Ψ}}_{k+1}. \end{array} \end{array}$$ (23)

Then, from the update rule of $y_{i}^{k+1}$ in Algorithm 1 together with Equations 10 and 11, we derive an expression for $z_{i}^{k}-x^{k}$:


$$\begin{array}{l} z_{i}^{k}-x^{k}=\frac{1}{\lambda }(y_{i}^{k}-y_{i}^{k+1})\\ \;=\frac{1}{\lambda }(w_{i}^{k}-w_{i}^{k+1})+\frac{\gamma }{\lambda }(\nabla f_{i}(w_{i}^{k})-\nabla f_{i}(w_{i}^{k+1}))\\ \;=\frac{1}{\lambda }(z_{i}^{k}-z_{i}^{k+1})+\frac{\gamma }{\lambda }(\nabla f_{i}(z_{i}^{k})-\nabla f_{i}(z_{i}^{k+1}))\\ \;+\frac{1}{\lambda }[(m_{i}^{k+1}+\gamma (\nabla f_{i}(z_{i}^{k+1})-\nabla f_{i}(w_{i}^{k+1})))\\ \;-(m_{i}^{k}+\gamma (\nabla f_{i}(z_{i}^{k})-\nabla f_{i}(w_{i}^{k})))]\\ \;=\frac{1}{\lambda }(z_{i}^{k}-z_{i}^{k+1})+\frac{\gamma }{\lambda }(\nabla f_{i}(z_{i}^{k})-\nabla f_{i}(z_{i}^{k+1}))+n_{i}^{k}, \end{array}$$ (24)

where $n_{i}^{k}$ is a composite error term involving the approximation errors $m_{i}^{k}$, $m_{i}^{k+1}$ and gradient differences. The subsequent analysis will control the impact of $n_{i}^{k}$ via its norm bound. It is defined as


$$\begin{array}{l} n_{i}^{k}=\frac{1}{\lambda }[(m_{i}^{k+1}+\gamma (\nabla f_{i}(z_{i}^{k+1})\\ \;-\nabla f_{i}(w_{i}^{k+1})))-(m_{i}^{k}+\gamma (\nabla f_{i}(z_{i}^{k})-\nabla f_{i}(w_{i}^{k})))], \end{array}$$

Its squared norm satisfies


$$\begin{array}{l} ||n_{i}^{k}|{|}^{2}=\frac{1}{\lambda^{2}}||m_{i}^{k+1}-m_{i}^{k}+\gamma \left(\nabla f_{i}\left(z_{i}^{k+1}\right)-\nabla f_{i}\left(w_{i}^{k+1}\right)\right)\\ \;+\gamma \left(\nabla f_{i}\left(w_{i}^{k}\right)-\nabla f_{i}\left(z_{i}^{k}\right)\right)|{|}^{2}\\ \;\leq \frac{2{\left(1+\gamma L\right)}^{2}}{\lambda^{2}}\left[||m_{i}^{k}|{|}^{2}+||m_{i}^{k+1}|{|}^{2}\right] \end{array}$$

By applying the *L*-smoothness of *f*_*i*_, the Young's inequality, and Equation 24, we obtain for any β_4_>0 that


$$\begin{array}{l} V^{k+1}\left(x^{k+1}\right)\leq V^{k}\left(x^{k}\right)+\frac{\left[\lambda \left(1+L\gamma \right)-2\right]}{2\lambda \gamma n}\underset{i\in S_{k}}{\sum }||z_{i}^{k+1}-z_{i}^{k}|{|}^{2}\\ \;+\frac{\gamma }{\lambda n}\underset{i\in S_{k}}{\sum }||\nabla f_{i}\left(z_{i}^{k+1}\right)-\nabla f_{i}\left(z_{i}^{k}\right)|{|}^{2}\\ \;+\frac{1}{\gamma n}\underset{i\in S_{k}}{\sum }\langle n_{i}^{k},\left(z_{i}^{k+1}-z_{i}^{k}\right)+\gamma \left(\nabla f_{i}\left(z_{i}^{k}\right)-\nabla f_{i}\left(z_{i}^{k+1}\right)\right)\rangle +{\text{Ψ}}_{k+1}\\ \;\left(by\;the\;L-smoothness\;of\;f_{i}\right)\\ \leq V^{k}\left(x^{k}\right)+\frac{\gamma L^{2}}{\lambda n}\underset{i\in S_{k}}{\sum }||z_{i}^{k+1}-z_{i}^{k}|{|}^{2}\\ \;+\frac{\left[\lambda \left(1+L\gamma \right)-2\right]}{2\lambda \gamma n}\underset{i\in S_{k}}{\sum }||z_{i}^{k+1}-z_{i}^{k}|{|}^{2}+{\text{Ψ}}_{k+1}\\ \;+\frac{1}{\gamma n}\underset{i\in S_{k}}{\sum }[\frac{1}{\beta_{4}}||n_{i}^{k}|{|}^{2}+2\beta_{4}||z_{i}^{k}-z_{i}^{k+1}|{|}^{2}+2\beta_{4}\gamma^{2}||\nabla f_{i}\left(z_{i}^{k}\right)\\ \;-\nabla f_{i}\left(z_{i}^{k+1}\right)|{|}^{2}]\\ \;\leq V^{k}\left(x^{k}\right)-\frac{\left[2-\lambda \left(1+L\gamma \right)-2L^{2}\gamma^{2}-4\lambda \beta_{4}\left(1+L^{2}\gamma^{2}\right)\right]}{2\lambda \gamma n}\\ \underset{i\in S_{k}}{\sum }||z_{i}^{k+1}-z_{i}^{k}|{|}^{2}\;+\frac{1}{\gamma \beta_{4}n}\underset{i\in S_{k}}{\sum }||n_{i}^{k}|{|}^{2}+{\text{Ψ}}_{k+1}\\ \leq V^{k}\left(x^{k}\right)-\frac{\left[2-\lambda \left(1+L\gamma \right)-2L^{2}\gamma^{2}-4\lambda \beta_{4}\left(1+L^{2}\gamma^{2}\right)\right]}{2\lambda \gamma n}\\ \underset{i\in S_{k}}{\sum }||z_{i}^{k+1}-z_{i}^{k}|{|}^{2}\\ \;+\frac{2{\left(1+\gamma L\right)}^{2}}{\gamma \beta_{4}\lambda^{2}n}\underset{i\in S_{k}}{\sum }\left[||m_{i}^{k}|{|}^{2}+||m_{i}^{k+1}|{|}^{2}\right]+{\text{Ψ}}_{k+1}. \end{array}$$ (25)

Next, leveraging the *L*-smoothness of *f*_*i*_ and assuming $\gamma \leq \frac{1}{L}$, we demonstrate the boundedness of *V*^*k*^(*x*^*k*^)


$$\begin{array}{l} V^{k}\left(x^{k}\right)=g\left(x^{k}\right)+\frac{1}{n}\overset{n}{\underset{i=1}{\sum }}[f_{i}\left(z_{i}^{k}\right)+\langle \nabla f_{i}\left(z_{i}^{k}\right),x^{k}-z_{i}^{k}\rangle \\ \;+\frac{1}{2\gamma }||x^{k}-z_{i}^{k}|{|}^{2}]\\ \;\geq g\left(x^{k}\right)+\frac{1}{n}\overset{n}{\underset{i=1}{\sum }}\left[f_{i}\left(x^{k}\right)-\frac{L}{2}||x^{k}-z_{i}^{k}|{|}^{2}+\frac{1}{2\gamma }||x^{k}-z_{i}^{k}|{|}^{2}\right]\\ \;\geq F\left(x^{k}\right)+\left(\frac{1}{2\gamma }-\frac{L}{2}\right)\frac{1}{n}\overset{n}{\underset{i=1}{\sum }}||x^{k}-z_{i}^{k}|{|}^{2}\\ \;\geq F^{*}. \end{array}$$

From Lemma 1, we have


$$\begin{array}{l} \frac{\lambda^{2}}{2\left(1+\beta_{1}\right)\left(\gamma^{2}L^{2}+1\right)}\sum_{i\in S_{k}}||x^{k}-z_{i}^{k}|{|}^{2}\leq \sum_{i\in S_{k}}[||z_{i}^{k+1}-z_{i}^{k}|{|}^{2}\\ \;+\frac{2}{\beta_{1}}\left(||m_{i}^{k+1}|{|}^{2}+||m_{i}^{k}|{|}^{2}\right)]. \end{array}$$ (26)

According to the sampling scheme, we consider the expectation of $\underset{i\in S_{k}}{\sum }||z_{i}^{k+1}-z_{i}^{k}|{|}^{2}$ with respect to $S_{k}$ conditioned on $A_{k-1}$. Combined with (26), this yields


$$\begin{array}{l} \begin{array}{l} 𝔼\left[\sum_{i\in S_{k}}||z_{i}^{k+1}-z_{i}^{k}|{|}^{2}|A_{k-1}\right]\\ \;=\sum_{S}\mathbb{P}\left(S_{k}=S\right)\sum_{i\in S}||z_{i}^{k+1}-z_{i}^{k}|{|}^{2}=\overset{n}{\underset{i=1}{\sum }}{\text{p}}_{i}||z_{i}^{k+1}-z_{i}^{k}|{|}^{2}\\ \geq \frac{\text{p}\lambda^{2}}{2\left(1+\beta_{1}\right)\left(\gamma^{2}L^{2}+1\right)}\overset{n}{\underset{i=1}{\sum }}||x^{k}-z_{i}^{k}|{|}^{2}\\ \;-\frac{2\text{p}}{\beta_{1}}\overset{n}{\underset{i=1}{\sum }}\left(||m_{i}^{k+1}|{|}^{2}+||m_{i}^{k}|{|}^{2}\right), \end{array} \end{array}$$ (27)

where **p** = min**p**_*i*_∈(0, 1], *i*∈[*n*]. By taking the conditional expectation of Equation 25 with respect to $S_{k}$ conditioned on $A_{k-1}$, and combining it with Equations 10, 21, 27 under the setting β_3_ = 1, we derive the following


$$\begin{array}{l} E[V^{k+1}(x^{k+1})|A_{k-1}]\\ \;\overset{\left(21\right)}{\leq }V^{k}(x^{k})+\frac{2{\left(1+\gamma L\right)}^{2}}{\gamma \beta_{4}\lambda^{2}n}\sum_{i=1}^{n}p_{i}[\|m_{i}^{k}{\|}^{2}+\|m_{i}^{k+1}{\|}^{2}]\\ \;-\frac{\left[2-\lambda (1+L\gamma )-2L^{2}\gamma^{2}-4\lambda \beta_{4}(1+L^{2}\gamma^{2})\right]}{2\lambda \gamma n}\\ E[\sum_{i\in S_{k}}{||z_{i}^{k+1}-z_{i}^{k}||}^{2}|A_{k-1}]\\ \;+\frac{2\varepsilon_{1}}{n\gamma }E[\sum_{i=1}^{n}{||e_{i}^{k}||}^{2}+\sum_{i=1}^{n}{||e_{i}^{k+1}||}^{2}]+\frac{\left(1+\gamma^{2}L^{2}\right)}{n\gamma }\\ \sum_{i=1}^{n}\left[(1-p_{i})\|m_{i}^{k}{\|}^{2}+p_{i}\|m_{i}^{k+1}{\|}^{2}\right]\\ \;(\text{by the definition of absolute compressor})\\ \overset{\left(27\right)}{\leq }V^{k}(x^{k})+\frac{2{\left(1+\gamma L\right)}^{2}}{\gamma \beta_{4}\lambda^{2}n}\sum_{i=1}^{n}p_{i}[\|m_{i}^{k}{\|}^{2}+\|m_{i}^{k+1}{\|}^{2}]\\ \;-\frac{p\lambda [2-\lambda (1+L\gamma )-2L^{2}\gamma^{2}-4\lambda \beta_{4}(1+L^{2}\gamma^{2})]}{4\gamma n(1+\beta_{1})(\gamma^{2}L^{2}+1)}\;\\ \;\sum_{i=1}^{n}{||x^{k}-z_{i}^{k}||}^{2}\\ \;+\frac{p[2-\lambda (1+L\gamma )-2L^{2}\gamma^{2}-4\lambda \beta_{4}(1+L^{2}\gamma^{2})]}{\lambda \gamma \beta_{1}n}\\ \;\sum_{i=1}^{n}\left(\|m_{i}^{k+1}{\|}^{2}+\|m_{i}^{k}{\|}^{2}\right)\\ \;+\frac{4\varepsilon_{1}}{\gamma }\nu^{2}+\frac{\left(1+\gamma^{2}L^{2}\right)}{n\gamma }\sum_{i=1}^{n}\left[(1-p_{i})\|m_{i}^{k}{\|}^{2}+p_{i}\|m_{i}^{k+1}{\|}^{2}\right]\;\\ \;\overset{\left(10\right)}{\leq }V^{k}(x^{k})-\frac{\pi }{2n}\sum_{i=1}^{n}{||x^{k}-z_{i}^{k}||}^{2}+\frac{4\varepsilon_{1}}{\gamma }\nu^{2}+\frac{1}{n}\sum_{i=1}^{n}(\delta_{1}(\varepsilon_{i}^{k}{)}^{2}\\ +\delta_{2}(\varepsilon_{i}^{k+1}{)}^{2}). \end{array}$$

To guarantee the descent property, let


$$\begin{array}{ll} \pi & =\frac{\text{p}\lambda \left[2-\lambda \left(1+L\gamma \right)-2L^{2}\gamma^{2}-4\lambda \beta_{4}\left(1+L^{2}\gamma^{2}\right)\right]}{2\gamma \left(1+\beta_{1}\right)\left(\gamma^{2}L^{2}+1\right)}>0. \end{array}$$

Then, we have


$$\begin{array}{l} 0<\lambda <\frac{\min\left\{\sqrt{4\beta_{4}+\frac{17}{16}}-\frac{1}{4},2\right\}}{4\beta_{4}+1}\;\mathrm{and}\\ 0<\gamma <\frac{\sqrt{{\left(1-\frac{\lambda }{4}\right)}^{2}-\lambda^{2}\beta_{4}\left(4\beta_{4}+1\right)}-\frac{\lambda }{4}}{L\left(2\lambda \beta_{4}+1\right)}. \end{array}$$

Theorem 1. Let $\left\{\left(y_{i}^{k},z_{i}^{k},x_{i}^{k},e_{i}^{k},x^{k}\right)\right\}$ be generated by Algorithm 1. Suppose that Assumptions 1 and 2 hold, for $0<\gamma <\frac{\sqrt{{\left(1-\frac{\lambda }{4}\right)}^{2}-\lambda^{2}\beta_{4}\left(4\beta_{4}+1\right)}-\frac{\lambda }{4}}{L\left(2\lambda \beta_{4}+1\right)}\;\mathrm{and}\;0<\lambda <\frac{\min\left\{\sqrt{4\beta_{4}+\frac{17}{16}}-\frac{1}{4},2\right\}}{4\beta_{4}+1}$, we have


$$\begin{array}{l} \begin{array}{l} \frac{1}{K}\overset{K-1}{\underset{k=0}{\sum }}𝔼\left[||G_{\gamma }\left(x^{k}\right)|{|}^{2}\right]\leq \frac{M_{1}}{K}\left(F\left(x^{0}\right)-F^{*}\right)\\ \;+\frac{1}{nK}\overset{K-1}{\underset{k=0}{\sum }}\overset{n}{\underset{i=1}{\sum }}\left[M_{2}{\left(\varepsilon_{i}^{k}\right)}^{2}+M_{3}{\left(\varepsilon_{i}^{k+1}\right)}^{2}\right]\\ \;+\frac{M_{4}}{K}\nu^{2}, \end{array} \end{array}$$ (28)

where


$$\begin{array}{l} M_{1}=\frac{4\left(1+\beta_{2}\right){\left(1+\gamma L\right)}^{2}}{\pi \gamma^{2}},\;M_{2}=\frac{\left(2\delta_{1}\beta_{2}+\pi \right)}{\beta_{2}}M_{1}\\ M_{3}=\delta_{2}M_{1},\;M_{4}=\frac{4\varepsilon_{1}K}{\gamma }M_{1}+\frac{4K}{n\gamma^{2}}, \end{array}$$

with ε_1_, β_2_>0, and π, δ_1_, δ_2_ defined in Lemma 3.

Proof. First, it follows from Lemma 3 that


$$\begin{array}{l} \overset{n}{\underset{i=1}{\sum }}||x^{k}-z_{i}^{k}|{|}^{2}\leq \frac{2n}{\pi }[V^{k}\left(x^{k}\right)-E\left[V^{k+1}\left(x^{k+1}\right)|A_{k-1}\right]\\ \;+\frac{4\varepsilon_{1}}{\gamma }\nu^{2}+\frac{1}{n}\overset{n}{\underset{i=1}{\sum }}\left(\delta_{1}{\left(\varepsilon_{i}^{k}\right)}^{2}+\delta_{2}{\left(\varepsilon_{i}^{k+1}\right)}^{2}\right)]. \end{array}$$

Combining the derived estimates and Lemma 2, we obtain


$$\begin{array}{l} \begin{array}{l} ||G_{\gamma }\left(x^{k}\right)|{|}^{2}\leq \frac{2{\left(1+\gamma L\right)}^{2}}{n\gamma^{2}}\overset{n}{\underset{i=1}{\sum }}[\left(1+\beta_{2}\right)||z_{i}^{k}-x^{k}|{|}^{2}\\ \;+\left(1+\frac{1}{\beta_{2}}\right)||m_{i}^{k}|{|}^{2}]+\frac{2}{n\gamma^{2}}\overset{n}{\underset{i=1}{\sum }}||e_{i}^{k-1}-e_{i}^{k}|{|}^{2}\\ \;\leq \frac{4\left(1+\beta_{2}\right){\left(1+\gamma L\right)}^{2}}{\pi \gamma^{2}}[V^{k}\left(x^{k}\right)\\ \;-𝔼\left[V^{k+1}\left(x^{k+1}\right)|A_{k-1}\right]]\\ \;+\frac{4\left(1+\beta_{2}\right){\left(1+\gamma L\right)}^{2}}{n\pi \gamma^{2}}\overset{n}{\underset{i=1}{\sum }}\left(\delta_{1}{\left(\varepsilon_{i}^{k}\right)}^{2}+\delta_{2}{\left(\varepsilon_{i}^{k+1}\right)}^{2}\right)\\ \;+\frac{2\left(1+\beta_{2}\right){\left(1+\gamma L\right)}^{2}}{n\gamma^{2}\beta_{2}}{\left(\varepsilon_{i}^{k}\right)}^{2}+\frac{2}{n\gamma^{2}}\overset{n}{\underset{i=1}{\sum }}||e_{i}^{k-1}-e_{i}^{k}|{|}^{2}\\ \;+\frac{16\left(1+\beta_{2}\right){\left(1+\gamma L\right)}^{2}\varepsilon_{1}}{\pi \gamma^{3}}\nu^{2}. \end{array} \end{array}$$ (29)

Taking the total expectation of $||G_{\gamma }\left(x^{k}\right)|{|}^{2}$ with respect to $A_{k}$, and by using the update of $e_{i}^{k}$ and the definition of the absolute compressor, we obtain the following result


$$\begin{array}{l} 𝔼\left[||G_{\gamma }\left(x^{k}\right)|{|}^{2}\right]\leq M_{1}\left(𝔼\left[V^{k}\left(x^{k}\right)\right]-𝔼\left[V^{k+1}\left(x^{k+1}\right)\right]\right)\\ \;+\frac{M_{2}}{n}\overset{n}{\underset{i=1}{\sum }}{\left(\varepsilon_{i}^{k}\right)}^{2}+\frac{M_{3}}{n}\overset{n}{\underset{i=1}{\sum }}{\left(\varepsilon_{i}^{k+1}\right)}^{2}+\frac{M_{4}}{K}\nu^{2}, \end{array}$$

where


$$\begin{array}{l} M_{1}=\frac{4(1+\beta_{2}){\left(1+\gamma L\right)}^{2}}{\pi \gamma^{2}},\;M_{2}=\frac{\begin{array}{c} 2(1+\beta_{2}){\left(1+\gamma L\right)}^{2}(4\delta_{1}\beta_{2}\\ +2\pi ) \end{array}}{\gamma^{2}\beta_{2}\pi }\\ M_{3}=\frac{4(1+\beta_{2}){\left(1+\gamma L\right)}^{2}\delta_{2}}{\pi \gamma^{2}},\;M_{4}=\frac{16(1+\beta_{2}){\left(1+\gamma L\right)}^{2}\varepsilon_{1}K}{\pi \gamma^{3}}\\ +\frac{4K}{n\gamma^{2}}, \end{array}$$

is four constants. Summing the inequality over *k* from 0 to *K*−1, and then scaling the resultant sum by $\frac{1}{K}$, we derive


$$\begin{array}{l} \begin{array}{l} \frac{1}{K}\overset{K-1}{\underset{k=0}{\sum }}𝔼\left[||G_{\gamma }\left(x^{k}\right)|{|}^{2}\right]\leq \frac{M_{1}}{K}\left(𝔼\left[V^{0}\left(x^{0}\right)\right]-E\left[V^{K}\left(x^{K}\right)\right]\right)\\ \;+\frac{1}{K}\overset{K-1}{\underset{k=0}{\sum }}[\frac{M_{2}}{n}\overset{n}{\underset{i=1}{\sum }}{\left(\varepsilon_{i}^{k}\right)}^{2}+\frac{M_{3}}{n}\overset{n}{\underset{i=1}{\sum }}{\left(\varepsilon_{i}^{k+1}\right)}^{2}\\ \;+\frac{M_{4}}{K}\nu^{2}]. \end{array} \end{array}$$ (30)

With the initial condition $z_{i}^{0}=x^{0}$, we obtain $V^{0}\left(x^{0}\right)=g\left(x^{0}\right)+\frac{1}{n}\overset{n}{\underset{i=1}{\sum }}f_{i}\left(z_{i}^{0}\right)=F\left(x^{0}\right)$. Together with the lower bound *E*[*V*^*k*+1^(*x*^*k*+1^)]≥F^*^, this implies that Equation 30 simplifies to


$$\begin{array}{l} \begin{array}{l} \frac{1}{K}\overset{K-1}{\underset{k=0}{\sum }}𝔼\left[||G_{\gamma }\left(x^{k}\right)|{|}^{2}\right]\leq \frac{M_{1}}{K}\left(F\left(x^{0}\right)-F^{*}\right)\\ \;+\frac{1}{nK}\overset{K-1}{\underset{k=0}{\sum }}\overset{n}{\underset{i=1}{\sum }}\left[M_{2}{\left(\varepsilon_{i}^{k}\right)}^{2}+M_{3}{\left(\varepsilon_{i}^{k+1}\right)}^{2}\right]\\ \;+\frac{M_{4}}{K}\nu^{2}, \end{array} \end{array}$$ (31)

which proves Equation 28.

Corollary 1. Suppose that Assumptions 1 and 2 hold, EF-Feddr (Algorithm 1) will find a ε-stationary point *x* such that $E||G_{\gamma }\left(x^{k}\right)||\leq \varepsilon$ in the following number of iterations


$$\begin{array}{l} K\geq \frac{M_{1}\left[F\left(x^{0}\right)-F^{*}\right]+\left(M_{2}+M_{3}\right)M+M_{4}\nu^{2}}{\varepsilon^{2}}, \end{array}$$

where *M*>0 is a constant, and *M*_1_, *M*_2_, *M*_3_, *M*_4_ are defined in Theorem 1. Consequently, the communication complexity is $K=O\left(\frac{1}{\varepsilon^{2}}\right)$.

Proof. As described in Tran-Dinh et al. (2021), the choice of accuracies $\varepsilon_{i}^{k}$ is constrained such that for a given constant *M*>0, $\frac{1}{n}\overset{K-1}{\underset{k=0}{\sum }}\overset{n}{\underset{i=1}{\sum }}{\left(\varepsilon_{i}^{k}\right)}^{2}\leq M$. Therefore,


$$\begin{array}{ll} \frac{1}{K}\overset{K-1}{\underset{k=0}{\sum }}𝔼\left[||G_{\gamma }\left(x^{k}\right)|{|}^{2}\right] & \leq \frac{\begin{array}{c} M_{1}\left(F\left(x^{0}\right)-F^{*}\right)+\left(M_{2}+M_{3}\right)M\\ +M_{4}\nu^{2} \end{array}}{K}. \end{array}$$ (32)

Consequently, to guarantee $E||G_{\gamma }\left(x^{k}\right)||\leq \varepsilon$, we have


$$\begin{array}{l} K\geq \frac{M_{1}\left[F\left(x^{0}\right)-F^{*}\right]+\left(M_{2}+M_{3}\right)M+M_{4}\nu^{2}}{\varepsilon^{2}}. \end{array}$$

Therefore, we can take $\left(K=\lfloor \frac{M_{1}[F(x^{0})-F^{*}]+(M_{2}+M_{3})M+M_{4}\nu^{2}}{\varepsilon^{2}}\rfloor =O(\frac{1}{\varepsilon^{2}})\;\right)$ as its lower bound.

## 5 Experiments

In the experiments, we evaluate EF-Feddr against Eco-FedSplit (Khirirat et al., 2022), Eco-FedProx (Khirirat et al., 2022), and FedDR (Tran-Dinh et al., 2021). In all compression-based baselines, the compression operator *C* denotes Top-*k* sparsification. For a fair comparison, we implement Eco-FedSplit, Eco-FedProx, and EF-Feddr on top of the FedDR framework. All experiments are conducted in TensorFlow (Abadi et al., 2016) on a cluster equipped with NVIDIA Tesla P100 (16 GB) GPUs. We next describe the datasets and models used in our study.

### 5.1 Non-IID datasets

We evaluate on both synthetic and real-world datasets: synthetic-(*l, s*), FEMNIST, and Shakespeare. Following prior studies (Caldas et al., 2018; Tran-Dinh et al., 2021), we generate synthetic-(*l, s*) with (*l, s*) = {(0, 0), (1, 1)}, where *l* controls the number of differing local models and *s* controls the degree of local data heterogeneity; larger *l* and *s* imply stronger non-IID heterogeneity. FEMNIST extends MNIST to 62 classes with over 800k samples; we use an 80%/20% train/test split and partition by writer, which naturally induces client-level heterogeneity. Shakespeare is a character-level language modeling corpus; we partition by user/play, so each client holds a distinct subset of texts (plays/scenes), yielding non-uniform label distributions across clients. In this context, the degree of non-IID-ness within each client's dataset is quantified by the number of classes present. Specifically, the Shakespeare dataset's non-IID-ness is delineated by the allocation of various plays' texts among clients. Each client is allocated a distinct subset of the corpus, which may include a varying number of plays and scenes. This results in a non-uniform distribution of text, where certain clients predominantly receive data from specific plays, whereas others obtain a more diverse range of content. Analogously, the FEMNIST dataset establishes non-IID-ness through the distribution of handwriting samples across different writers. Each client's dataset comprises samples from a subset of writers, thereby leading to variability in handwriting styles and features among clients. The datasets and model configurations used in our experiments are summarized in Table 2, which outlines their key statistical characteristics.

**Table 2:** Dataset and model characteristics for federated training.

**Dataset**	**Client participation**	**Samples**	**Model**	**Parameters**
Synthetic-(0, 0)	1/3	75,349	ANN	2,282
Synthetic-(1, 1)	1/3	75,349	ANN	2,282
FEMNIST	1/4	18,345	CNN	214,370
Shakespeare	10/143	517,106	LSTM	817,872

### 5.2 Models and hyper-parameters selection

We use a fully connected network with a 60-32-10 architecture and train it for 200 communication rounds with a learning rate of 0.01 on all synthetic datasets. At each round, 10 out of 30 clients are sampled. To evaluate the algorithm's performance with an increased number of clients, we further extended the Synthetic-(1,1) setup from the original 30 clients to 90 clients while preserving the statistical characteristics defined by the (*l, s*) parameters. The data generation process maintained the same non-IID partition pattern and per-client data distribution profile as the original setup. The client sampling ratio was kept constant at 1/3 (that is, selecting 30 out of 90 clients per round). Eco-FedSplit applies error-compensated compression to FedSplit, and Eco-FedProx does so to FedProx. To study an image classification problem on FEMNIST, we employ artificial neural networks (ANN) consisting of two fully connected layers. The first layer has 128 neurons followed by a ReLU activation function, and the second layer has 62 neurons followed by a softmax activation function for classification. In this experiment, we sample 50 clients out of 200 to perform updates at each communication round for all the above-mentioned algorithms. The model used for FEMNIST is trained for 200 communication rounds in total with an optimal learning rate of 0.003. Consistent with prior research (Li et al., 2020), our approach to character-level prediction in the Shakespeare dataset utilizes a recurrent neural network (RNN) architecture. Specifically, we deploy a two-layer stacked LSTM classifier, each layer comprising 256 hidden units. Each input sequence is structured to include 80 characters, which are initially embedded into an eight-dimensional space prior to LSTM processing. The model subsequently generates a 62-class softmax distribution over the character vocabulary for each training instance. The training regimen involves a total of 50 communication rounds. An optimal learning rate of 0.08 is determined for the four operator-splitting-based federated learning algorithms employed in this study. Parameters for each algorithm such as α∈(0, 2) and η∈[1, 1, 000] for FedDR, μ∈[0.001, 1] for Eco-FedProx, and λ∈(0, 2) and γ∈[1, 1, 000] for EF-Feddr are tuned from a large range of values. For each dataset, we pick the most suitable parameters for each algorithm.

### 5.3 Comparison of methods

**Figure 1:**
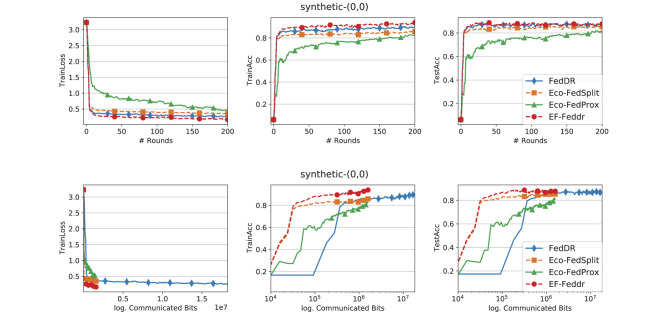
Convergence performance of different methods on the synthetic-(0, 0) dataset with Top-*k* and participation rate *p* = 0.3.

**Figure 2:**
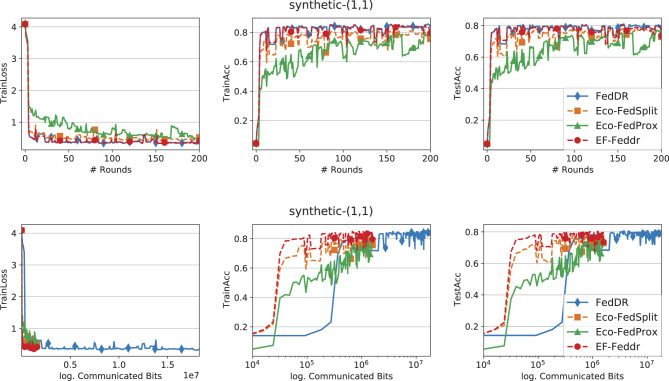
Convergence performance of different methods on the synthetic-(1, 1) dataset with Top-*k*, participation rate *p* = 0.3, and *N* = 30 total clients.

**Figure 3:**
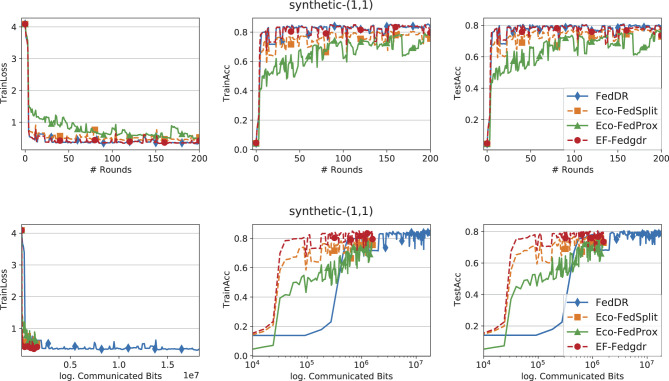
Convergence performance of different methods on the synthetic-(1, 1) dataset with Top-*k*, participation rate *p* = 0.3, and *N* = 90 total clients.

**Figure 4:**
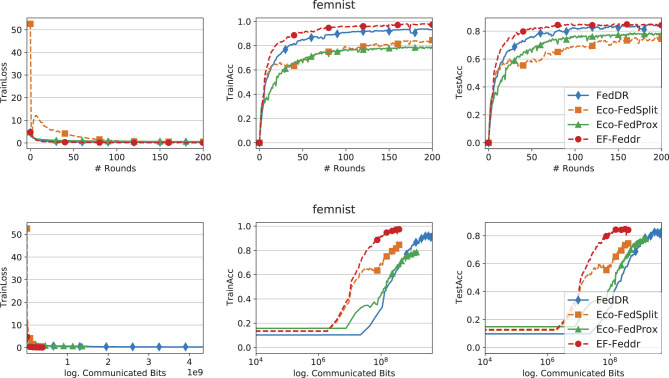
Convergence performance of different methods on the FEMNIST dataset with Top-*k* and participation rate *p* = 0.3.

**Figure 5:**
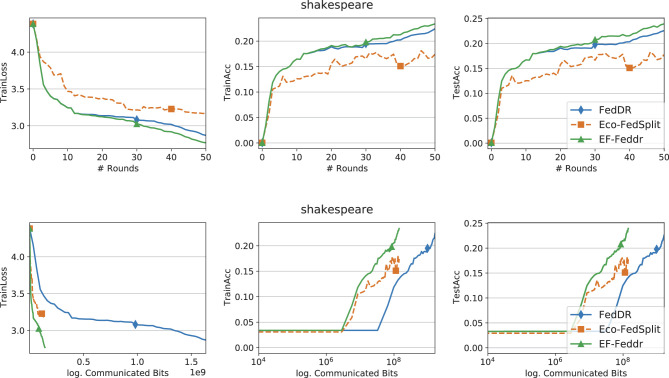
Convergence performance of different methods on the Shakespeare dataset with Top-*k* and participation rate *p* = 0.3.

Figures 1–3 report training loss/accuracy and test accuracy vs. communication rounds and communication cost on the synthetic datasets; Figure 4 shows the same on FEMNIST. A key observation is that expanding the total number of clients does not substantially degrade the performance of EF-Feddr. Experimental results under the scaled setting (Figure 3) confirm this: the algorithm maintains nearly identical convergence speed and final accuracy compared to the original 30-client scenario (Figure 2). Across heterogeneous settings, EF-Feddr consistently outperforms the baselines. On FEMNIST, EF-Feddr reaches 80.5% test accuracy at round 50, whereas Eco-FedSplit attains 74.5% only at round 200. Within 200 rounds, EF-Feddr improves accuracy by 12.97% and 7.93% over Eco-FedSplit and Eco-FedProx, respectively. On synthetic-(0, 0), EF-Feddr exceeds the two baselines by 3.88% and 8.40%; on synthetic-(1, 1), by 7.20% and 3.29%. On Shakespeare, Figure 5 shows EF-Feddr also surpasses two Douglas–Rachford splitting-based FL algorithms: Eco-FedSplit and FedDR. As shown in Table 3, EF-Feddr requires 18.64%–85.41% less runtime and 48.03%–93.18% less communication than baseline methods to achieve the same target test accuracy of 60% on synthetic and 70% on FEMNIST. Specifically, on FEMNIST, it meets this target in only 17 communication rounds (8.29 min), significantly outperforming competitors like Eco-FedSplit. These substantial reductions in overhead are consistently observed across the synthetic datasets. Additionally, EF-Feddr achieves a substantial reduction in communication costs without compromising performance relative to the uncompressed FedDR.

**Table 3:** Efficiency comparison on synthetic-(1, 1) and femnist datasets.

**Method**	**Synthetic-**(1, 1) **(60% accuracy)**			**FEMNIST (70% accuracy)**		
	**Rounds**	**Time (min)**	**Comm (MB)**	**Rounds**	**Time (min)**	**Comm (GB)**
Eco-FedProx	29	6.58	0.21	61	19.72	0.24
Eco-FedSplit	12	2.85	0.09	98	48.72	0.172
FedDR	5	1.18	0.44	33	15.95	0.67
EF-Feddr	4	0.96	0.03	17	8.29	0.059

### 5.4 Effect of the relaxation parameter

**Figure 6:**
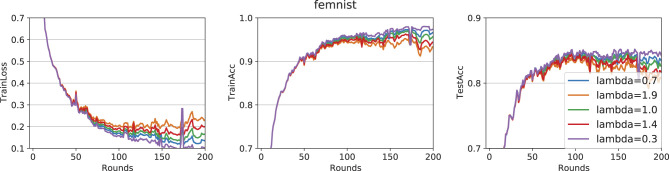
EF-Feddr on FEMNIST with relaxation parameter λ analysis.

Figure 6 examines the effect of the relaxation parameter λ over 200 iterations. Empirically, the best convergence is observed at λ = 0.3. Consistent with prior findings on FL adaptations of Douglas–Rachford splitting, choosing 0 <λ <1 often leads to faster convergence than the classical (unrelaxed) variant.

## 6 Discussion

This study presents EF-Feddr, a communication-efficient federated learning algorithm that combines error-compensated compression with Douglas–Rachford splitting. The method's robustness is demonstrated across controlled synthetic and real-world benchmarks, yet we recognize that extreme heterogeneity, such as single-class clients, remains a challenging frontier. Furthermore, while our experiments simulate realistic constraints (partial participation, compression), fully asynchronous updates and dynamic network conditions warrant further study in real deployments.

Recent advances in behavior-based threat hunting (Bhardwaj et al., 2022), IoT firmware security assessment (Bhardwaj et al., 2023), and energy-efficient proactive fault tolerance in cloud environments (Talwar et al., 2021) provide complementary perspectives for building reliable and secure federated systems. While this study focuses on optimization efficiency under non-IID and communication constraints, these studies collectively point toward an integrated “Optimization + System + Security” paradigm for future research. Specifically, they motivate investigations into client behavior profiling for attack detection, trusted execution at the edge, and proactive fault-tolerant scheduling, all of which are essential for deploying robust and efficient federated learning in real-world, dynamic environments. Furthermore, to strengthen the generalizability of our findings, future studies will also include evaluations on a wider variety of datasets, encompassing diverse domains, scales, and heterogeneity patterns, thereby providing a more comprehensive assessment of the algorithm's practical applicability.

## 7 Conclusion

In this study, we introduced EF-Feddr, a communication-efficient algorithm for non-convex federated learning that leverages the Douglas–Rachford splitting method, error feedback compression, and a relaxation strategy. EF-Feddr improves communication efficiency while preserving solution accuracy. Both theoretical analysis and empirical experiments demonstrated that EF-Feddr substantially reduces the number of bits transmitted from clients to the server compared with uncompressed FedDR. In terms of solution accuracy, EF-Feddr performs comparably to the uncompressed FedDR. Building on the Douglas–Rachford envelope, we established convergence guarantees and analyzed the communication complexity of EF-Feddr under mild assumptions. Extensive experiments further confirmed that our method significantly outperforms existing state-of-the-art approaches in non-IID settings.

## Data Availability

Publicly available datasets were analyzed in this study. This data can be found here: arXiv preprint arXiv:1812.01097.
